# *Burkholderia thailandensis* Isolated from Infected Wound, Southwest China, 2022

**DOI:** 10.3201/eid3005.230743

**Published:** 2024-05

**Authors:** Jin Li, Jishan Tan, Xingyun Xiong, Qiu Zhong, Weiping Lu

**Affiliations:** Chongqing Medical University Affiliated Dazu Hospital, Chongqing, China (J. Li);; Daping Hospital of Army Medical University, Chongqing (J. Li, Q. Zhong, W. Lu);; General Hospital of Western Theater Command, Chengdu, China (J. Tan);; Dazhu County People’s Hospital, Dazhou, Sichuan, China (X. Xiong)

**Keywords:** *Burkholderia thailandensis*, bacteria, infected wound, China

## Abstract

We report a clinical isolate of *Burkholderia thailandensis* 2022DZh obtained from a patient with an infected wound in southwest China. Genomic analysis indicates that this isolate clusters with *B. thailandensis* BPM, a human isolate from Chongqing, China. We recommend enhancing monitoring and surveillance for *B. thailandensis* infection in both humans and livestock.

*Burkholderia thailandensis* is a member of the *Burkholderia pseudomallei* complex and is generally considered nonpathogenic (*1*). Initially identified in Thailand, *B. thailandensis* was distinguished from *B. pseudomallei* by its ability to assimilate arabinose (*2*). Similar to *B. pseudomallei*, *B. thailandensis* is frequently found in soil and water, especially in rice paddies (*3*,*4*). Although invasive infections caused by *B. thailandensis* are rare worldwide, recent reports have documented cases of suppurative infections, such as wound infections, cellulitis, and tissue abscesses (*5*,*6*). Previously, we identified a strain of *B. thailandensis* BPM that caused a human infection in Chongqing, southwest China (*7*). In this study, we report another clinical isolate of *B. thailandensis* that we obtained in Dazhu, Sichuan, southwest China, from an infected wound resulting from a cut inflicted by a farm tool in 2022.

A 61-year-old male farmer who had untreated type 2 diabetes mellitus reported a 1-month history of pain and swelling in his left knee. He had injured the middle toe of his left foot with a plow a month earlier, and redness, swelling, and pain developed below the left knee joint. Despite a week of antimicrobial treatment at a local community health center, his symptoms did not improve. In December 2022, the patient began to experience weakness in his right lower limb, and he later fell, sustaining an injury to his left lower limb. During this period, he experienced lower-limb weakness and exhibited symptoms related to the central nervous system. He sought care and was admitted to the orthopedics department of Dazhu County People’s Hospital (Dazhou, China) for treatment of a left lower-limb injury and a left-foot diabetic foot infection. However, because his central nervous system symptoms worsened, he was transferred to the neurology department and receive a diagnosis of osteomyelitis and demyelinating disease. *B. thailandensis* strain 2022DZh was obtained from a deep-tissue specimen during surgical debridement of the infected wound on the left foot (Appendix Figure 1). The initial empirical antimicrobial therapy consisted of cefradine. However, the hospital laboratory tested the isolate 2022DZh using the VITEK 2 COMPACT system (bioMérieux, https://www.biomerieux.com) and identified it as *B. pseudomallei*, leading to a switch to intravenous meropenem treatment (Appendix Table 1). Despite treatment with meropenem, the patient’s condition continued to deteriorate; he died 3 days after applying for discharge. 

Subsequently, the isolate 2022DZh was submitted to the laboratory for confirmatory identification. Results of arabinose assimilation testing of isolate 2022DZh by API 20NE system (bioMérieux) were positive, consistent with the biochemical characteristics of *B. thailandensis* (Appendix Figure 2). We extracted DNA from the isolate 2022DZh for confirmation and further characterization. We performed 16S rRNA gene sequencing of 2022DZh using nucleotide BLAST (https://blast.ncbi.nlm.nih.gov/Blast.cgi), revealing 100% similarity with *B. thailandensis* BPM (Appendix Figure 3). The nucleotide sequences of the 2 chromosomes of isolate 2022DZh are >99.5% consistent with those of *B. thailandensis* E264 and *B. thailandensis* E254 (Appendix Table 2). We conclusively identified isolate 2022DZh as *B. thailandensis* based on our phenotypic and molecular data. We deposited the genome sequences of *B. thailandensis* strain 2022DZh into GenBank (accession nos. CP141809.1 and CP141811.1).

For phylogenetic analysis, we compared the genome of *B. thailandensis* 2022DZh with a reference panel of publicly available *Burkholderia* species genomes (Appendix Table 3). The single-copy gene phylogenetic tree analysis indicated that *B. thailandensis* 2022DZh clusters with *B. thailandensis* BPM (Figure, panel A). The results of average nucleotide identity revealed that *B. thailandensis* 2022DZh also clusters with *B. thailandensis* BPM; genome identity was 99.85%. However, when compared with *B. pseudomallei* BPC006, genome identity was 92.31% (Figure, panel B), consistent with the commonly used 95% threshold for distinguishing species. Through multilocus sequence type analysis (https://pubmlst.org/organisms/burkholderia-pseudomallei) (*8*), we determined that *B. thailandensis* 2022DZh and *B. thailandensis* BPM both belong to sequence type 76. In addition, there appears to be no known epidemiologic link between *B. thailandensis* 2022DZh and *B. thailandensis* BPM; they are geographically separated by a significant distance of ≈200 km (Appendix Table 3). 

**Figure:**
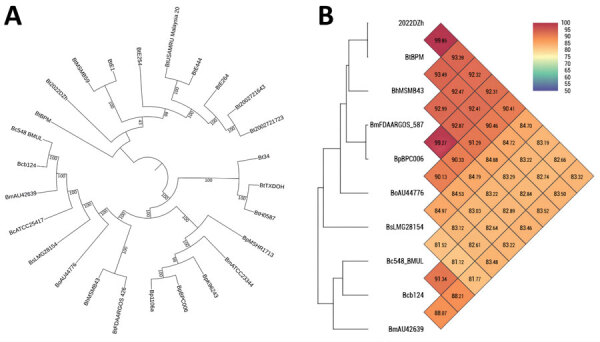
Analysis of the single-copy gene phylogenetic tree and average nucleotide identity for genomes of *Burkholderia thailandensis* 2022DZh from a patient in Dazhu, Sichuan, China, and other isolates from *Burkholderia* species. A) Single-copy gene phylogenetic tree created using the genomes of *B. thailandensis* 2022DZh and 25 other isolates from various *Burkholderia* species. B) Average nucleotide identity heatmap developed using genomes of *B. thailandensis* 2022DZh and 9 other isolates from various *Burkholderia* species.

One limitation of this study is that we did not attempts to identify related isolates of *B. thailandensis* from environmental samples in this region of southwest China. Further studies are needed to identify the primary molecular mechanisms underlying the pathogenicity of *B. thailandensis* 2022DZh and to determine its molecular and evolutionary relationships with other strains of *B. thailandensis* (*9*).

In conclusion, our findings underscore that *B. thailandensis* can cause serious infections, and clinical practitioners should be aware of this type of infection (*10*). Therefore, we strongly recommend enhancing monitoring and surveillance for *B. thailandensis* infection in both humans and livestock.

Appendix Additional information about *Burkholderia thailandensis* isolated from infected wound, China, 2022
