# Supplementary material for: Burkholderia thailandensis Isolated from Infected Wound, Southwest China, 2022
Source: Emerg Infect Dis. 2024 May;30(5):1055–7. doi: 10.3201/eid3005.230743 (PMC11060469; doi:10.3201/eid3005.230743)
Supplement: Appendix — Additional information about Burkholderia thailandensis isolated from infected wound, China, 2022 [file 23-0743-Techapp-s1.pdf]

Article DOI: http://doi.org/10.3201/eid3005.230743

EID cannot ensure accessibility for supplementary materials supplied by authors. Readers who have difficulty accessing supplementary content should contact the authors for assistance.

Burkholderia thailandensis Isolated from Infected Wound, Southwest China, 2022

Appendix

Appendix Table 1. Antibiotic susceptibility testing results of *B. thailandensis* 2022DZh

Antimicrobial	MIC	Interpretation
Amoxicillin/clavulanate	≤4	S
Ceftazidime	2	S
Imipenem	≤1	S
Tetracycline	4	S
Doxycycline	≤0.5	S
Trimethoprim/sulfamethoxazole	40	S

Appendix Table 2. The Nucleotide Sequence Database (NT) identified strain 2022DZh as *B. thailandensis**

Query_name	Query_ length	Query_ start	Query_ end	Hit_name	Hit_description	Hit_length	Hit_start	Hit_end	Aln_length	Identity
chr1	3804194	627187	941171	CP008785.1	*Burkholderia thailandensis* E264 chromosome 1, complete sequence	3986340	1022844	1336839	314053	99.9032010520517
chr2	2860001	831328	941171	CP004382.1	*Burkholderia thailandensis* E254 chromosome 2, complete sequence	2870750	2145935	2478653	332805	99.874100449212
plasmid1	215545	135181	157561	CP013426.1	*Burkholderia* sp. MSMB0856 plasmid pMSMB0856, complete sequence	249331	8541	30930	22395	98.0442062960482

*The NT database is the Nucleotide Sequence Database, and the link is http://www.ncbi.nlm.nih.gov

Appendix Table 3. Genomes of additional *Burkholderia* species isolates used for single-copy gene phylogenetic tree analysis and average nucleotide identity (ANI) analysis in the study of *B. thailandensis* 2022DZh

Isolate	Abbreviation	Origin	Year	Source	MLST	GenBank accession no.
B. thailandensis H0587	BtH0587	USA	1997	Clinical (Human)	101	NZ_CP004089.1; NZ_CP004090.1
B. thailandensis BPM	BtBPM	China	2013	Clinical (Human)	76	NZ_CP050020.1; NZ_CP050021.1
B. thailandensis E264	BtE264	Thailand	1994	Environmental (soil)	80	NZ_CP008785.1; NZ_CP008786.1
B. thailandensis E254	BtE254	Thailand	1992	Environmental (soil)	345	NZ_CP004381.1; NZ_CP004382.1
B. thailandensis E444	BtE444	Thailand	2002	Environmental (soil)	79	NZ_CP004117.1; NZ_CP004118.1
B. thailandensis MSMB59	BtMSMB59	Australia	2006	Environmental (soil)	669	NZ_CP004385.1; NZ_CP004386.1
B. thailandensis USAMRU Malaysia 20	BtUSAMRU Malaysia 20	Malaysia	2015	Unknown	80	NZ_CP004383.1; NZ_CP004384.1
B. thailandensis 34	Bt34	USA	2002	Unknown	73	NZ_CP010017.1; NZ_CP010018.1
B. thailandensis 2002721723	Bt2002721723	USA	2013	Clinical (Human)	80	NZ_CP004098.1; NZ_CP004097.1
B. thailandensis 2002721643	Bt2002721643	USA	2002	Unknown	80	NZ_CP009601.1; NZ_CP009602.1
B. thailandensis E1	BtE1	Papua New Guinea	1995	Environmental (soil)	669	NZ_LOXF00000000.1
B. thailandensis TXDOH	BtTXDOH	USA	2003	Clinical (Human)	101	NZ_ABBD00000000.1
B. thailandensis FDAARGOS_426	BtFDAARGOS_426	Malaysia	2017	Environmental (soil)	1023	NZ_CP023499.1; NZ_CP023498.1
B. pseudomallei K96243	BpK96243	Thailand	1993	Clinical (Human)	10	NZ_CP009538.1; NZ_CP009537.1
B. pseudomallei BPC006	BpBPC006	China	2008	Clinical (Human)	70	NZ_CP003781.1; NZ_CP003782.1
B. pseudomallei 1106a	Bp1106a	Thailand	2008	Clinical (Human)	70	NZ_CP008758.1; NZ_CP008759.1
B. pseudomallei MSHR1713	BpMSHR1713	Australia	2003	Clinical (Human)	131	NZ_CP111138.1; NZ_CP111139.1
B. pseudomallei MSHR1046	BpMSHR1046	Australia	2000	Clinical (Human)	131	NZ_CP111140.1; NZ_CP111141.1

Isolate	Abbreviation	Origin	Year	Source	MLST	GenBank accession no.
B. mallei ATCC23344	BmATCC23344	USA	1942	Clinical (Human)	40	NZ_CP124608.1; NZ_CP124607.1
B. singularis LMG 28154	BsLMG 28154	Canada	2003	Clinical (Human)	166	NZ_FXAN01000000
B. oklahomensis AU44776	BoAU44776	USA	2022	Clinical (Human)	1772	NZ_JAUJRD000000000.1
B. cepacia b124	Bcb124	Bangladesh	2017	Clinical (Human)	140	NZ_SNSI01000000
B. cenocepacia 548_BMUL	Bc548_BMUL	USA	2016	Clinical (Human)	162	NZ_JVCQ01000000
B. multivorans AU42639	BmAU42639	USA	2020	Clinical (Human)	79	NZ_JAHPLO010000000
B. cocovenenans ATCC25417	BcATCC25417	USA	2012	Environmental (leaves)	101	NZ_JPGG00000000.1
B. mallei FDAARGOS_587	BmFDAARGOS_587	USA	1961	Environmental (Horse)	40	NZ_RKJX00000000.1
B. humptydooensis MSMB43	BhMSMB43	Australia	1995	Environmental (Water)	318	NZ_AJXB00000000.1

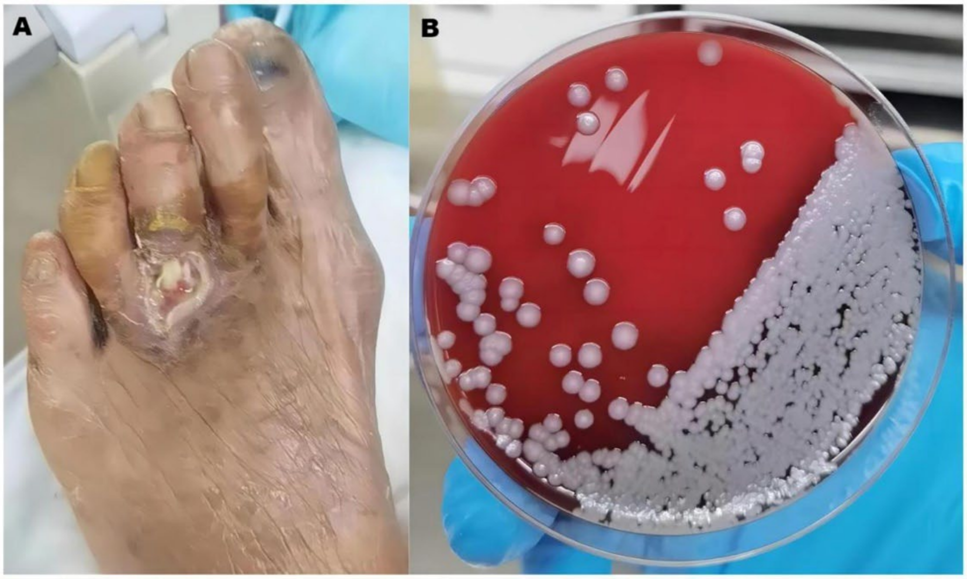

Appendix Figure 1. Morphologic features of left-foot diabetic foot infection caused by *B. thailandensis* 2022DZh in a patient in Dazhu, Sichuan, China**.** A) Result of surgical debridement

of a wound in the 61-year-old male patient. B) Colony morphology of *B. thailandensis* 2022DZh from the patient on a Columbia blood plate.

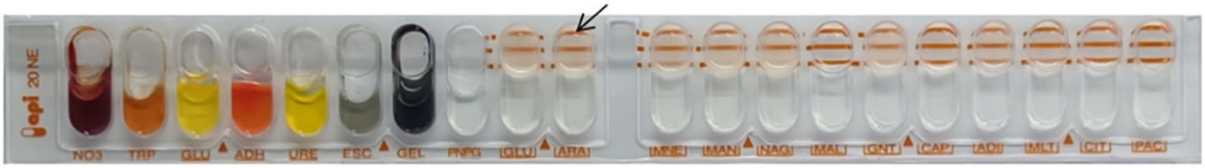

Appendix Figure 2. The biochemical profiles of the API 20NE system, including arabinose assimilation, identified isolate 2022DZh as *B. thailandensis.*

Sequences producing significant alignments Download Select columns Show 100

select all *100 sequences selected* GenBank Graphics Distance tree of results MSA Viewer

Description	Scientific Name	Max Score	Total Score	Query Cover	E value	Per. Ident	Acc. Len	Accession
Burkholderia thailandensis strain BPM chromosome 2, complete sequence	Burkholderia thailandensis	2542	2542	100%	0.0	100.00%	2875717	CP050021.1
Burkholderia thailandensis strain BPM chromosome 1, complete sequence	Burkholderia thailandensis	2542	7626	100%	0.0	100.00%	3725540	CP050020.1
Burkholderia thailandensis strain FDAARGOS_242 chromosome 1, complete sequence	Burkholderia thailandensis	2542	7606	100%	0.0	100.00%	3801254	CP022217.1
Burkholderia thailandensis strain FDAARGOS_242 chromosome 2, complete sequence	Burkholderia thailandensis	2542	2542	100%	0.0	100.00%	2852057	CP022216.1
Burkholderia thailandensis strain FDAARGOS_237 chromosome 1, complete sequence	Burkholderia thailandensis	2542	7618	100%	0.0	100.00%	3819771	CP020390.1
Burkholderia thailandensis strain MSMB59 chromosome 2, complete sequence	Burkholderia thailandensis	2542	2542	100%	0.0	100.00%	2822193	CP013408.1
Burkholderia thailandensis strain MSMB59 chromosome 1, complete sequence	Burkholderia thailandensis	2542	7626	100%	0.0	100.00%	3917337	CP013407.1
Burkholderia thailandensis 2002721643 chromosome II, complete sequence	Burkholderia thailandensis 2002721643	2542	2542	100%	0.0	100.00%	2914772	CP009602.1
Burkholderia thailandensis 2002721643 chromosome I, complete sequence	Burkholderia thailandensis 2002721643	2542	7626	100%	0.0	100.00%	3808029	CP009601.1
Burkholderia thailandensis E254 chromosome 2, complete sequence	Burkholderia thailandensis E254	2542	2542	100%	0.0	100.00%	2870750	CP004382.1
Burkholderia thailandensis E254 chromosome 1, complete sequence	Burkholderia thailandensis E254	2542	7620	100%	0.0	100.00%	3805980	CP004381.1
Burkholderia thailandensis MSMB59 chromosome 2, complete sequence	Burkholderia thailandensis MSMB59	2542	2542	100%	0.0	100.00%	2823176	CP004386.1
Burkholderia thailandensis MSMB59 chromosome 1, complete sequence	Burkholderia thailandensis MSMB59	2542	7620	100%	0.0	100.00%	3916324	CP004385.1
Burkholderia thailandensis E264 chromosome 2, complete sequence	Burkholderia thailandensis E264	2542	5076	100%	0.0	100.00%	2735759	CP008786.1
Burkholderia thailandensis E264 chromosome 1, complete sequence	Burkholderia thailandensis E264	2542	5076	100%	0.0	100.00%	3986340	CP008785.1
Burkholderia thailandensis USAMRU Malaysia #20 chromosome 2, complete sequence	Burkholderia thailandensis USAMRU ...	2542	2542	100%	0.0	100.00%	2821713	CP004384.1
Burkholderia thailandensis USAMRU Malaysia #20 chromosome 1, complete sequence	Burkholderia thailandensis USAMRU ...	2542	7615	100%	0.0	100.00%	3862646	CP004383.1
Burkholderia thailandensis E444 chromosome 2, complete sequence	Burkholderia thailandensis E444	2542	2542	100%	0.0	100.00%	2852101	CP004118.1
Burkholderia thailandensis E444 chromosome 1, complete sequence	Burkholderia thailandensis E444	2542	7618	100%	0.0	100.00%	3799595	CP004117.1
Burkholderia thailandensis 2002721723 chromosome 2, complete sequence	Burkholderia thailandensis 2002721723	2542	2542	100%	0.0	100.00%	2914718	CP004098.1
Burkholderia thailandensis 2002721723 chromosome 1, complete sequence	Burkholderia thailandensis 2002721723	2542	7626	100%	0.0	100.00%	3662415	CP004097.1
Burkholderia thailandensis strain AW34-19p chromosome 2, complete sequence	Burkholderia thailandensis	2542	2542	100%	0.0	100.00%	2826723	CP068710.1
Burkholderia thailandensis strain AW34-19p chromosome 1, complete sequence	Burkholderia thailandensis	2542	7484	100%	0.0	100.00%	3830943	CP068709.1
Burkholderia thailandensis E264 chromosome I, complete sequence	Burkholderia thailandensis E264	2542	7626	100%	0.0	100.00%	3809201	CP000086.1
Burkholderia thailandensis E264 16S ribosomal RNA, partial sequence	Burkholderia thailandensis E264	2536	2536	100%	0.0	99.93%	1529	NR_074312.2
Burkholderia thailandensis E264 chromosome II, complete sequence	Burkholderia thailandensis E264	2536	2536	100%	0.0	99.93%	2914771	CP000085.1
Burkholderia thailandensis strain FDAARGOS_241 chromosome 2, complete sequence	Burkholderia thailandensis	2534	2534	100%	0.0	99.93%	2821560	CP022215.1
Burkholderia thailandensis strain FDAARGOS_237 chromosome 2, complete sequence	Burkholderia thailandensis	2534	2534	100%	0.0	99.93%	2853041	CP020389.1
Burkholderia thailandensis strain BD10-00323 16S ribosomal RNA, partial sequence	Burkholderia thailandensis	2534	2534	99%	0.0	100.00%	1459	NR_118629.1
Burkholderia thailandensis strain BD11-00023 16S ribosomal RNA gene, partial seque...	Burkholderia thailandensis	2534	2534	99%	0.0	100.00%	1465	KF444905.1
Burkholderia thailandensis strain BD11-00022 16S ribosomal RNA gene, partial seque...	Burkholderia thailandensis	2534	2534	99%	0.0	100.00%	1457	KF444904.1
Burkholderia thailandensis E264 16S ribosomal RNA, partial sequence	Burkholderia thailandensis E264	2531	2531	99%	0.0	100.00%	1541	NR_116051.1
Burkholderia thailandensis strain 2002721627 16S ribosomal RNA gene, partial seque...	Burkholderia thailandensis	2531	2531	99%	0.0	100.00%	1488	AY268182.1
Burkholderia thailandensis strain FDAARGOS_241 chromosome 1, complete sequence	Burkholderia thailandensis	2529	7554	100%	0.0	99.85%	3862564	CP022214.1

Appendix Figure 3. The 16S rRNA gene sequencing of strain 2022DZh using nucleotide BLAST.
